# Characterization of Interplay Between Autophagy and Ferroptosis and Their Synergistical Roles on Manipulating Immunological Tumor Microenvironment in Squamous Cell Carcinomas

**DOI:** 10.3389/fimmu.2021.739039

**Published:** 2022-02-04

**Authors:** Lijie Chen, Xing Niu, Xue Qiao, Sai Liu, Hongmei Ma, Xueqing Shi, Xuemei He, Ming Zhong

**Affiliations:** ^1^ Department of Stomatology, Xiang’an Hospital of Xiamen University, School of Medicine, Xiamen University, Xiamen, China; ^2^ Department of Oral Histopathology, School and Hospital of Stomatology, China Medical University, Liaoning Province Key Laboratory of Oral Disease, Shenyang, China; ^3^ Department of Central Laboratory, School and Hospital of Stomatology, China Medical University, Liaoning Province Key Laboratory of Oral Disease, Shenyang, China

**Keywords:** squamous cell carcinomas, ferroptosis, autophagy, crosstalk, tumor microenvironment, immunity, drug resistance, prognosis

## Abstract

**Objective:**

Squamous cell carcinomas (SCCs) with shared etiology, histological characteristics, and certain risk factors represent the most common solid cancers. This study reports the crosstalk between autophagy and ferroptosis at the molecular level in SCCs, and their roles on the immunological tumor microenvironment (TME) of SCCs.

**Methods:**

In this study, the connections between autophagy and ferroptosis were characterized in SCCs by analyzing the associations between autophagy- and ferroptosis-related genes in mRNA expression and prognosis, protein-protein interactions and shared signaling pathways. Autophagy potential index (API) and ferroptosis potential index (FPI) of each tumor were quantified for reflecting autophagy and ferroptosis levels *via* principal-component analysis algorithm. Their synergistical roles on TME, immunity, drug resistance and survival were systematically analyzed in SCCs.

**Results:**

There were close connections between autophagy and ferroptosis at the mRNA and protein levels and prognosis. Both shared cancer-related pathways. The API and FPI were separately developed based on prognostic autophagy- and ferroptosis-related genes. A high correlation between API and FPI was found in SCCs. Their interplay was distinctly associated with favorable prognosis, enhanced sensitivity to chemotherapy drugs (Sunitinib, Gefitinib, Vinblastine and Vorinostat), an inflamed TME and higher likelihood of response to immunotherapy in SCCs.

**Conclusion:**

This study is the first to ﻿provide a comprehensive analysis of the interplay between autophagy and ferroptosis and their synergistical roles on manipulating the immunological TME in SCCs. These findings indicated that the induction of autophagy and ferroptosis combined with immunotherapy might produce synergistically enhanced anti-SCCs activity.

## Introduction

Squamous cell carcinomas (SCCs) represent the most common solid cancers (1). SCCs arise from epithelial tissues of the aerodigestive or genitourinary tracts, which are commonly detected in head and neck, esophagus, lung, and cervix (2). SCCs across different body sites share overlapping etiology, histopathological characteristics [such as the presence of keratin pearls, tonofilament bundles, hemidesmosomes and desmosomes (3)] and specific risk factors (such as smoking, drinking and human papillomavirus infection) (4). Previous the Cancer Genome Atlas (TCGA) research has uncovered that SCCs exhibit similar molecular patterns such as somatic mutations, copy number variations, abnormal pathways, and tumor microenvironment (TME) that differ from other cancer types (2, 5, 6). With surgery, radio- and chemotherapy as the standard of care for most SCCs, the treatment of SCCs is complex and has undergone considerable advancement in the last decade (7). Especially, treatment with immune checkpoint inhibitors (ICIs) such as anti-programmed death-1 (anti-PD-1), anti-programmed death ligand-1 (anti-PD-L1), and/or anti-cytotoxic T lymphocyte-associated antigen-4 (anti-CTLA-4) has been applied to SCCs, which can result in impressive response rates and durable disease remission in clinical trials (8–10). However, only in a subset of patients respond to ICI therapy (11).

Autophagy is an evolutionarily conserved cellular process, which may degrade various biological molecules and organelles through lysosome-dependent degradation pathway (12). Ferroptosis is a novel form of programmed cell death, which is driven by iron accumulation and lipid peroxidation (13). Recent research has revealed the role of autophagy in driving cells towards ferroptosis (14). Meanwhile, activation of autophagy is required for the induction of ferroptosis (12). The crosstalk between autophagy and ferroptosis decide cell fate through activating comprehensive signaling pathways and affecting gene expression programs (15). Growing evidence suggests that interplay of autophagy and ferroptosis exerts a key role in antitumor immunity (16), tumor suppression (17) and drug resistance (18), etc. However, the mechanism of the crosstalk between autophagy and ferroptosis in SCCs remains largely ill-defined. Uncovering when and how to modulate their interplay utilizing therapeutic strategies against SCCs depends on the in-depth understanding of the connections between autophagy and ferroptosis (18). Unraveling the interplay between autophagy and ferroptosis may not only elucidate fundamental mechanistic insights into SCCs, but also provide novel therapeutic targets for the treatment of SCCs. We hypothesize that appropriate combinations of potent drugs that specifically activate autophagy and ferroptosis with ICIs might achieve better treatment effects. Therefore, this study specifically and comprehensively characterized the interplay between ferroptosis and autophagy in SCCs and their synergistical roles on immunity, TME, drug resistance and prognosis of SCCs.

## Materials and Methods

### Patients and Datasets

**Figure 1:**
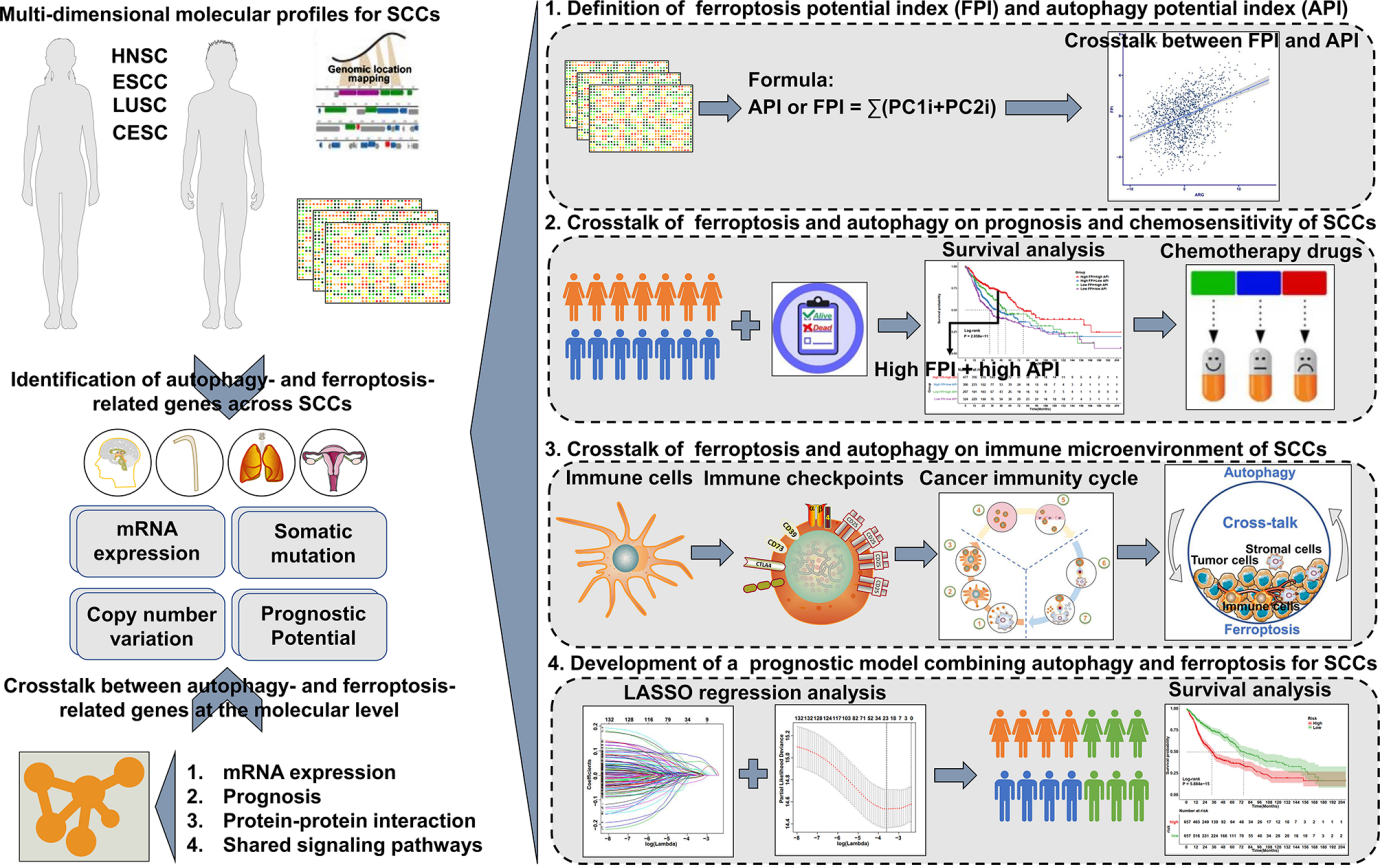
Overview of the study design.

**Figure 1** shows the workflow of this study. RNA sequencing (RNA-seq) data (fragments per kilobase of transcript per million mapped reads (FPKM) values) and clinical information of SCCs including head and neck squamous cell carcinoma (HNSC; n=500), lung squamous cell carcinoma (LUSC; n=501), cervical squamous cell carcinoma (CESC; n=241), and esophageal squamous cell carcinoma (ESCC; n=81) were acquired from TCGA (http://cancergenome.nih.gov) database *via* the Genomic Data Commons (GDC, https://portal.gdc.cancer.gov/). Then, FPKM values were converted into TPM values. Microarray datasets including gene expression profiling of GSE17710/LUSC (N=56) (19), GSE44001/CESC (N=300) (20), GSE65858/HNSC (N=270) (21) were employed from the Gene Expression Omnibus (GEO; http://www.ncbi.nlm.nih.gov/geo/) database. Batch effects from non-biological technical biases were corrected with the “ComBat” algorithm of sva package (version 3.42.0) (22). Ferroptosis- and autophagy-related genes were collected according to published literature (**Supplementary Table 1**). The locations of ferroptosis- and autophagy-related genes on human chromosomes were separately plotted by employing Rcircos package (version 1.2.1) (23). Genomic mutation data of SCCs [somatic mutation and copy number variation (CNV)] were also obtained from TCGA database. Mutation status was analyzed and visualized by maftools package (version 2.10.0) (24).

### Computational Models of Ferroptosis and Autophagy Levels Among SCCs

A ferroptosis and autophagy scoring scheme was developed to quantify ferroptosis and autophagy levels in each specimen with principal component analysis (PCA). Survival analysis of ferroptosis- and autophagy-related genes was separately performed utilizing univariate Cox regression analysis. The expression profiles of the genes with p<0.05 were extracted to carried out PCA, and principal component 1 and 2 were extracted and acted as the signature score. Like previous studies (25, 26), the ferroptosis potential index (FPI) and autophagy potential index (API) were separately defined: API or FPI = ∑(PC1_i_+PC2_i_), where i represents the expression of ferroptosis- or autophagy-related genes.

### Protein-Protein Interaction (PPI) Analysis

Interactions between ferroptosis- and autophagy-related genes were analyzed through the STRING online database (version: 11.0; https://string-db.org/) (27). A PPI network was generated and displayed by Cytoscape software (version: 3.7.2) (28).

### Survival Analysis

Kaplan–Meier curves for overall survival (OS), disease-free interval (DFI), disease-free survival (DFS), disease-specific survival (DSS) and progression-free interval (PFI) were plotted to compare the survival time differences. P-values were calculated with log-rank tests. Time-dependent receiver-operating characteristic (ROC) curve analysis was carried out using survivalROC package (version 1.0.3). The area under the ROC curve (AUC) was determined to assess the prognostic performance.

### Estimation of Tumor Microenvironment (TME)

Estimation of STromal and Immune cells in MAlignant Tumours using Expression data (ESTIMATE) may infer the tumor cellularity and tumor purity based on unique properties of the transcriptional profiles (29). Through ESTIMATE algorithm, immune and stromal scores were determined to estimate the levels of infiltrating immune and stromal cells as well as tumor purity. Tumor tissues with abundant immune cell infiltration indicate a higher immune score and lower level of tumor purity. Through the single-sample gene-set enrichment analysis (ssGSEA) algorithm, the enrichment scores of 16 immune cells and 13 immune functions for each sample were estimated based on the expression of marker genes of tumor-infiltrating immune cells (TIICs) that were obtained from Bindea et al. utilizing gene set variation analysis (GSVA) package [version 1.42.0 (30)]. The expression of human leukocyte antigen (HLA) genes, immune checkpoints and immunomodulators (including major histocompatibility complex (MHC) molecules, receptors, chemokines, and immunostimulatory factors) (31) was also quantified in each sample (**Supplementary Table 2**).

### Quantification of Immune Response Predictors

T cell dysfunction and exclusion (TIDE) (http://tide.dfci.harvard.edu/) algorithm was employed to characterize tumor immune evasion mechanism, including dysfunction of tumor infiltration cytotoxic T lymphocytes (CTLs) and exclusion of CTLs by immunosuppressors (32). Tumor mutation burden (TMB) for each sample was quantified according to mutation frequency with number of variants/the length of exons (33). The cancer immunity cycle includes release of cancer cell antigens (step 1), cancer antigen presentation (step 2), priming and activation (step 3), trafficking of immune cells to tumors (step 4), infiltration of immune cells into tumors (step 5), recognition of cancer cells by T cells (step 6), and killing of cancer cells (step 7) (**Supplementary Table 3**) (34). The activities of these steps were assessed with ssGSEA based on the gene expression of each sample (35).

### Prediction of Chemosensitivity

Sensitivity to chemotherapy drugs for each specimen was predicted by the Genomics of Drug Sensitivity in Cancer (GDSC, https://www.cancerrxgene.org/) database (36). Drugs including cisplatin, paclitaxel, gemcitabine, sorafenib, sunitinib, gefitinib, vinblastine and vorinostat were selected. The half-maximal inhibitory concentration (IC50) values were determined by ridge regression analysis using pRRophetic package (37).

### Gene Set Variation Analysis (GSVA)

GSVA package was employed for estimating the activity of pathways with a non-parametric and unsupervised method (38). The gene sets of “c2.cp.kegg.v7.2.symbols” were acquired from the Molecular Signatures Database (MSigDB) (39).

### Acquirement of mRNA Expression-Based Stemness Index (mRNAsi)

Cancer stemness of SCCs was quantified as described by Malta et al. (40). The mRNAsi of SCCs was calculated with one-class logistic regression machine learning algorithm and expressed with β values ranging from 0 (no gene expression) to 1 (complete gene expression).

### Identification of Autophagy- and Ferroptosis-Related Genes

Autophagy- and ferroptosis-related genes between high FPI + high API group and others group were screened by limma package (version 3.50.0) (41). Genes with |fold-change| >1.5 and false discovery rate (FDR) <0.05 were considered statistically significant. Gene Ontology (GO) and Kyoto Encyclopedia of Genes and Genomes (KEGG) enrichment analysis of the autophagy- and ferroptosis-related genes were performed using clusterProfiler package (version 4.2.0) (42). Terms with FDR <0.05 were significantly enriched.

### Connectivity Map (CMap) Analysis

The autophagy- and ferroptosis-related genes were used to query the CMap database (https://clue.io/) (43). Compounds with p<0.05 were considered as potential therapeutic drugs for ferroptosis and autophagy based on gene expression signatures. Furthermore, the mode of action (MoA) of these compounds was analyzed.

### Development of an Autophagy- and Ferroptosis-Related Prognostic Model

Univariate cox regression analysis was performed to screen prognostic autophagy- and ferroptosis-related genes with p <0.05. Least absolute shrinkage and selection operator (LASSO) regression method was applied for finding out the optimal candidate variables with glmnet package (version 4.1-3) (44). The optimal values of the penalty parameter lambda were determined by ten-fold cross‐validation. The risk score of each patient was calculated based on the expression and coefficient of candidate autophagy- and ferroptosis-related genes. The formula of the risk score was as follows: $\text{risk score}=\Sigma_{i=1}^{n}(\text{coefi}\times \text{Expri})$ , where Expri indicates the expression of each gene for patient i, and coefi indicates the coefficient of gene i. The patients were equally stratified into high‐ and low-risk groups. Kaplan-Meier curves of OS were performed between two groups. Time‐dependent ROC curves were plotted to determine the AUCs of OS using survivalROC package.

### Cell Culture and Treatment

Human SCCs cell lines (KYSE410 and KYSE450) were purchased from ATCC (Manassas, VA, USA). Cells were maintained in RPMI-1640 (#PM150120; Procell, Wuhan, China) plus 10% fetal bovine serum (FBS; #SH30084.03; Hyclone, South Logan, UT, USA), 100 units/mL penicillin, 100 μg/mL streptomycin. Rapamycin (#ab120224; Abcam, Cambridge, MA, USA), Erastin (#ab209693; Abcam, Cambridge, MA, USA) and Gefitinib (Iressa, AstraZeneca, Macclesfield, UK) were dissolved in dimethyl sulfoxide (DMSO; Sigma, St. Louis, MO, USA) as well as stored at -20°C. To activate autophagy or ferroptosis, cells were administrated with 0.1 μM Rapamycin or Erastin for 16 h.

### 3-[4,5-Dimethylthiazol-2-yl]-2, 5-Diphenyltetrazoliumbromide (MTT) Assays

Cell viability was conducted with MTT assays. Cells were seeded onto 96-well plates (1 × 10^3^ cells/well). Following 12 h of culture, cells were pre-treated with 0.1 μM Rapamycin or 10 μM Erastin for 16 h. Thereafter, cells were administrated with different concentrations of gefitinib (0, 0.01, 0.1, 1, 2, 3, 6 and 10 μM) for another 24 h. Cells were then stained with 20 μl MTT (5 mg/ml; #M5655-1G; Sigma-Aldrich, St. Louis, MO, USA) for 4 h at 37°C. Then, culture medium was removed as well as 150 μl DMSO was added. Viable cells were measured at 490 nm absorbance. Half inhibitory concentration (IC50) values were calculated with dose-response curves using GraphPad Prism software (version 8.0.1).

### Western Blotting

Cell lysates were prepared with RIPA lysis buffer (#P0013B; Beyotime, Shanghai, China) plus protease inhibitors. Protein concentration was measured through BCA kit (#P0009; Beyotime, Shanghai, China) accordance with the manufacturer’s instructions. Equal amount of protein was separated *via* SDS-PAGE electrophoresis and transferred onto PVDF membrane (Millipore, Billerica, MA). Membrane was incubated with antibodies against LC3 (1:1000; #14600-1-AP; Proteintech, Wuhan, China), ATG-5 (1:500; #10181-2-AP; Proteintech, Wuhan, China), ATG-7 (1:500; #10088-2-AP; Proteintech, Wuhan, China), FTH1 (1:1000; #3998S; Cell Signaling Technology, Danvers, MA, USA), GXP4 (1:1000; #67763-1-lg; Proteintech, Wuhan, China) and β-actin (1:5000; #60008-1-lg; Proteintech, Wuhan, China) overnight at 4°C. Thereafter, horseradish peroxide-conjugated goat anti-rabbit (1:5000; #SA00001-2; Proteintech, Wuhan, China) or anti-mouse (1:5000; #SA00001-1; Proteintech, Wuhan, China) secondary antibodies were utilized for immunostaining for 1 h at room temperature, followed by exposure to ECL reagent (#K-12043-D10; Wuhan Juneng Yitong Biological Co., Ltd., Wuhan, China). Images were acquired through ChemiDoc™XRS+ Gel imaging system (Bio-Rad, Hercules, CA, USA).

### 5-Ethynyl-2’-Deoxyuridine (EdU) Staining

Cells were incubated with RPMI-1640 medium plus 50 μM EdU (#C0078S; Beyotime, Beijing, China) at 37°C for 2 h. After being washed twice with PBS, cells were fixed with 50 μL 4% paraformaldehyde (#E672002; Sangon Biotech, Shanghai, China) for 30 min, neutralized with 50 μL 2 mg/mL glycine solution as well as permeabilized with 100 μL 0.5% Triton X-100. Thereafter, cells were incubated with 100 μL 1 × Apollo dye at room temperature for 30 min, followed by incubation with 100 μL Hoechst 33342 for 30 min. Images were acquired under a BX53 fluorescence microscope (Olympus, Japan).

### Transwell Assays

Invasion assays were conducted in 24-well transwell cell chamber coated with 30 μl Matrigel (#356234; BD Biocoat, USA). 3 × 10^5^ indicated cells were seeded onto the coated filters while the bottom chamber was filled with 600 μl complete culture medium. Following incubation for 48 h at 37°C, invasive cells were stained with crystal violent (#C0121; Beyotime, Shanghai, China). The migration assays were performed through a similar method without coating with Matrigel.

### Statistical Analysis

All the statistical analysis was executed by R software (version 4.0.1) and GraphPad Prism software (version 8.0.1). Each experiment was independently repeated three times. The Kolmogorov-Smirnov normality test was carried out to confirm if datasets followed a Gaussian distribution for each comparison. If the data were Gaussian, parametric test was carried out (unpaired student’s test, one-way ANOVA or Pearson correlation). If the data were non-Gaussian, nonparametric test was performed (Wilcoxon rank test or Spearman correlation). P <0.05 indicated statistical significance.

## Results

### Landscape of Genetic Variation of Autophagy- and Ferroptosis-Related Genes in SCCs

**Figure 2:**
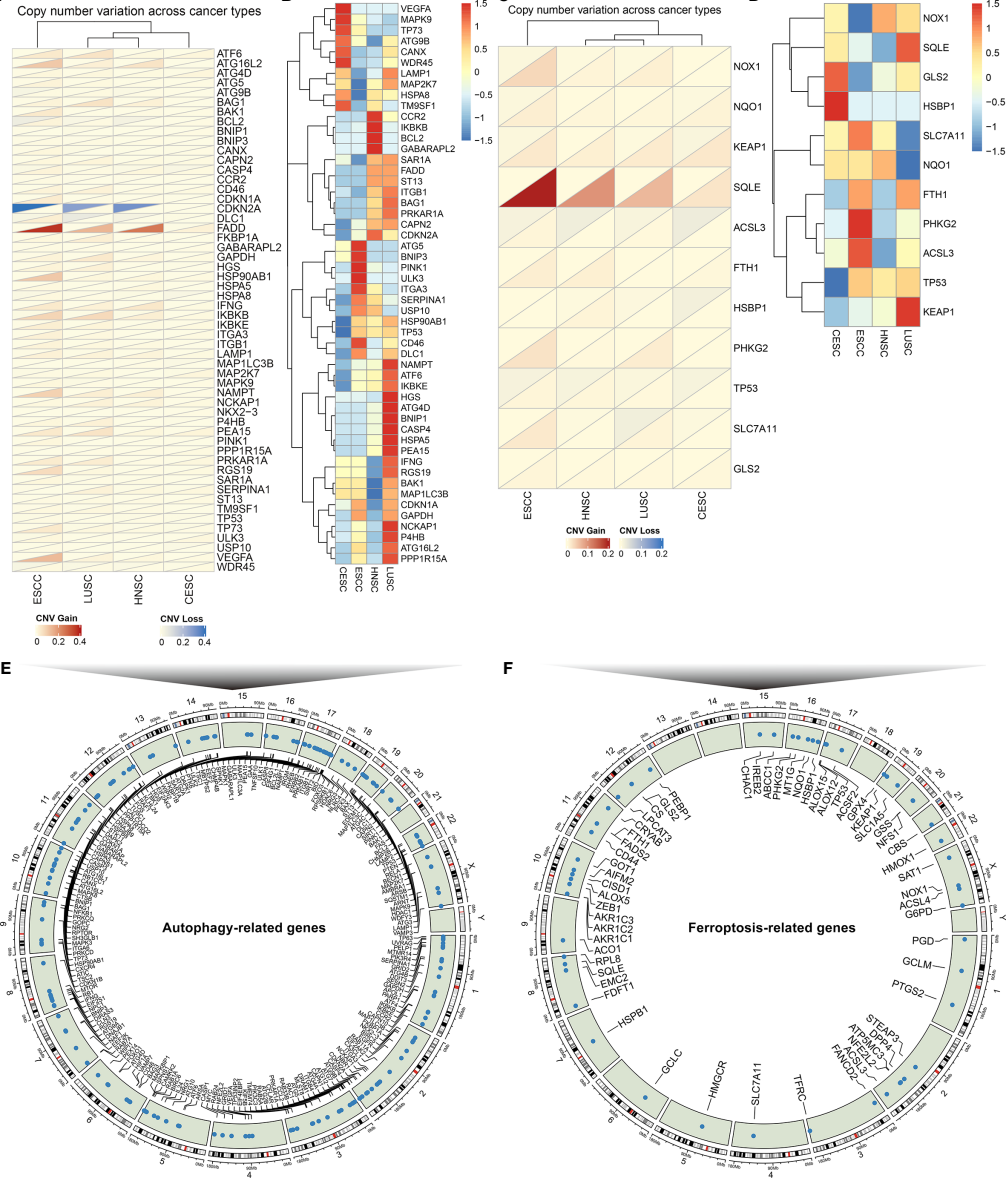
The landscape of genetic alterations of autophagy- and ferroptosis-related genes in SCCs. **(A)** The CNV frequency of autophagy-related genes across SCCs. Red: the gain frequency; blue: the loss frequency. **(B)** The SNP frequency of autophagy-related genes across SCCs. **(C)** The CNV frequency of ferroptosis-related genes in SCCs. **(D)** The SNP frequency of ferroptosis-related genes across SCCs. **(E)** The location of autophagy-related genes on 23 chromosomes. **(F)** The location of ferroptosis-related genes on 23 chromosomes.

Totally, 222 autophagy- and 60 ferroptosis-related genes were investigated in SCCs that integrated HNSC, LUSC, CESC and ESCC datasets (**Supplementary Figure 1**). We firstly determined the prevalence of CNV mutations of autophagy-related genes in SCCs. CNV mutations were less frequent in CESC while CNV amplification was prevalent in ESCC (**Figure 2A**). Particularly, CDKNA2A showed widespread CNV loss and FADD displayed widespread CNV amplification in ESCC, LUSC and HNSC. Further analysis of somatic mutation frequency displayed a prevalent somatic mutation in autophagy-related genes (**Figure 2B**). The incidence of CNV variations and somatic mutations of ferroptosis-related genes was also summarized in SCCs. The investigation of CNV mutation revealed a widespread occurrence, especially SQLE amplification in ESCC and ACSL3 loss in CESC (**Figure 2C**). Further analysis revealed a prevalent frequency of somatic mutations of ferroptosis-related genes in SCCs (**Figure 2D**). The somatic mutations of each SCC were summarized in **Supplementary Figure 2**. Collectively, we did not investigate any prominent effect on different kinds of mutations according to different tissue origins, indicating that SCCs exhibited similar CNV and somatic mutation patterns of autophagy- and ferroptosis-related genes. The locations of autophagy- and ferroptosis-related genes on chromosomes were shown in **Figures 2E, F**. The above analysis revealed that autophagy and ferroptosis were precisely regulated at multiple layers in SCCs.

### Crosstalk Between Autophagy and Ferroptosis in SCCs at the Molecular Level

**Figure 3:**
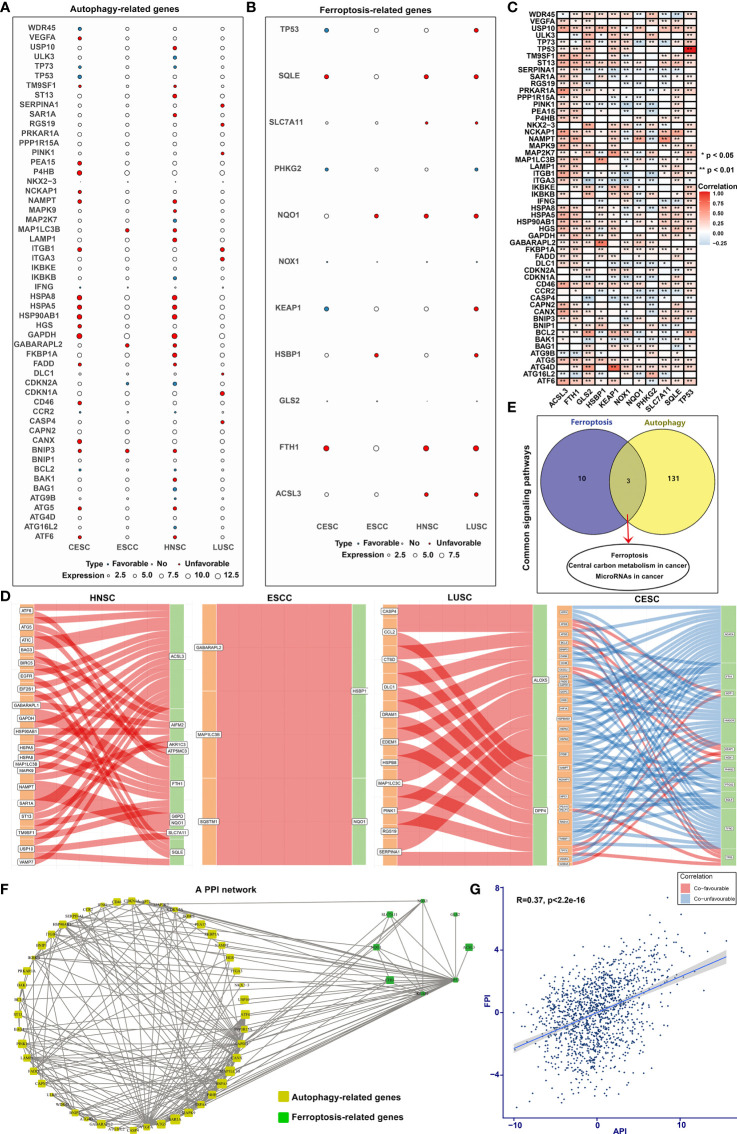
Cross-talk between autophagy and ferroptosis in SCCs at the molecular level. **(A)** Bubble diagram showing correlations between autophagy-related genes and prognosis of SCCs using univariate cox regression analysis. Blue bubbles represented positive correlations with favorable survival outcomes and red bubbles represented positive correlations with unfavorable prognosis. The size of the bubble showed the expression level of each gene. **(B)** Correlations between ferroptosis-related genes and prognosis of SCCs. **(C)** Heatmap showing correlations between autophagy- and ferroptosis-related genes across SCCs at the mRNA level. Positive correlation was marked with red and negative correlation with blue. *p<0.05; **p<0.01. **(D)** Alluvial diagram for the shared effects of autophagy- and ferroptosis-related genes on prognosis of SCCs. **(E)** Venn diagram showing the common signals enriched by autophagy- and ferroptosis-related genes. **(F)** The PPI network of the interactions between autophagy- and ferroptosis-related genes. **(G)** Correlations between API and FPI in SCCs using Spearman test.

Univariate Cox regression analysis was applied to ascertain the relationship between the mRNA expression of autophagy- or ferroptosis-related genes and the prognosis of SCCs patients (**Figures 3A, B**; **Supplementary Figures 3** **–** **5**). Some autophagy- or ferroptosis-related genes served as protective factors of the prognosis of SCCs, others were considered risk factors. Pearson correlation analysis was employed to investigate mutual regulation between these prognostic genes in SCCs. We found that autophagy-related genes presented remarkable correlations to the ferroptosis-related genes in terms of mRNA levels (**Figure 3C**) and prognosis (**Figure 3D**). KEGG enrichment analysis of autophagy- and ferroptosis-related genes was conducted, respectively. Ferroptosis, central carbon metabolism in cancer and microRNAs in cancer were collectively enriched by two sets of genes (**Figure 3E**). The close interactions of autophagy- and ferroptosis-related genes were also illustrated in the PPI network (**Figure 3F**). The API (**Supplementary Table 4**) and FPI (**Supplementary Table 5**) were separately calculated to quantify autophagy and ferroptosis levels in individual tumors based on the autophagy- and ferroptosis-related genes that could significantly impact prognosis of SCCs patients. There was a mutual regulation between FPI and API in SCCs (**Figure 3G**). The above results indicated that the crosstalk of autophagy and ferroptosis may play critical roles in SCCs initiation and progression.

### The Roles of Autophagy on Outcomes, TME, Response to Immunotherapy and Chemosensitivity in SCCs

**Figure 4:**
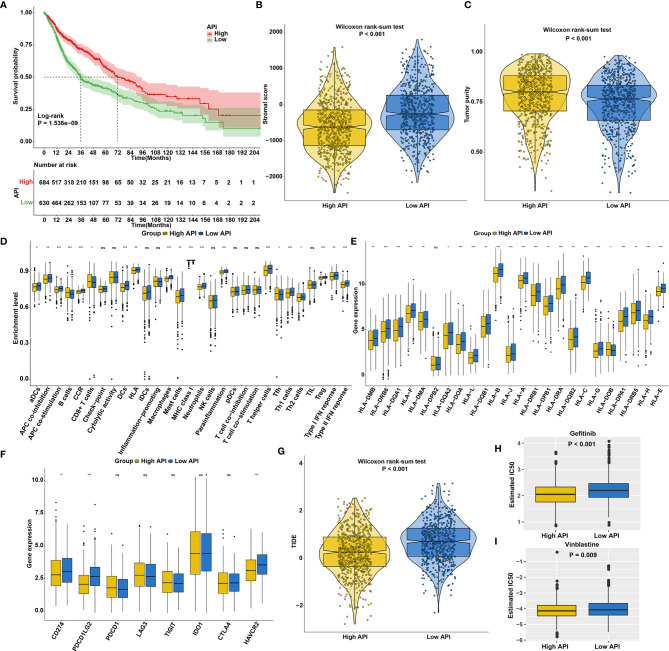
The roles of autophagy on outcomes, TME, response to immunotherapy and chemosensitivity in SCCs. **(A)** Kaplan-Meier curves of overall survival for SCCs patients with high and low API. P value was determined with log-rank test. **(B)** Differences in stromal scores between high and low API groups in SCCs cohort. **(C)** Differences in tumor purity between high and low API groups. **(D)** Differences in the enrichment levels of immune cells and immune functions between two groups. **(E)** Differences in the expression levels of HLA genes between two groups. **(F)** Differences in the expression levels of immune checkpoints between two groups. **(G)** Differences in TIDE scores between two groups. **(H, I)** Differences in estimated IC50 values of **(H)** Gefitinib and **(I)** Vinblastine between two groups. Ns, not significant; *p<0.05; **p<0.01; ***p<0.001.

The SCCs patients were stratified into high and low API groups based on the median value of API. Prognoses analysis for the two groups suggested the remarkably prominent survival advantage in high API (**Figure 4A**). Stromal score represents the percentage of stromal cells in the TME. High API was characterized by decreased stromal score (**Figure 4B**). Tumor purity, which reflects the proportion of cancer cells in the tumor tissue, is associated with a favorable clinical outcome of SCCs (45). High API showed the significantly increased tumor purity than low API (**Figure 4C**). Further analysis aimed at immunological role of autophagy in SCCs. We found that high API was significantly associated with low infiltration of immune cells (**Figure 4D**), low HLA expression (**Figure 4E**) and low immune checkpoint expression (**Figure 4F**). Based on the spatial distribution of cytotoxic immune cells in the TME, tumors may be categorized into immune-inflamed (also described as hot tumors), immune-excluded, and immune-desert phenotypes (46). Immune-excluded and immune-desert tumors are also named as “cold tumors”. These data suggested that SCCs with high API may lack innate immunity or innate antitumor immune features and autophagy could lead to “cold tumors”. TIDE score exhibits the high accuracy in predicting cancer immunotherapy response (32). Our analysis showed that TIDE score was significantly decreased in high API samples (**Figure 4G**). These findings indicated that autophagy might be involved in the immunosuppression of SCCs. Chemotherapy resistance is the principal limitation of clinical oncology. High API samples were more sensitive to Gefitinib (**Figure 4H**) and Vinblastine (**Figure 4I**), indicating that autophagy could mediate resistance to chemotherapy drugs.

### The Roles of Ferroptosis on Prognosis, TME, Response to Immunotherapy and Chemosensitivity in SCCs

**Figure 5:**
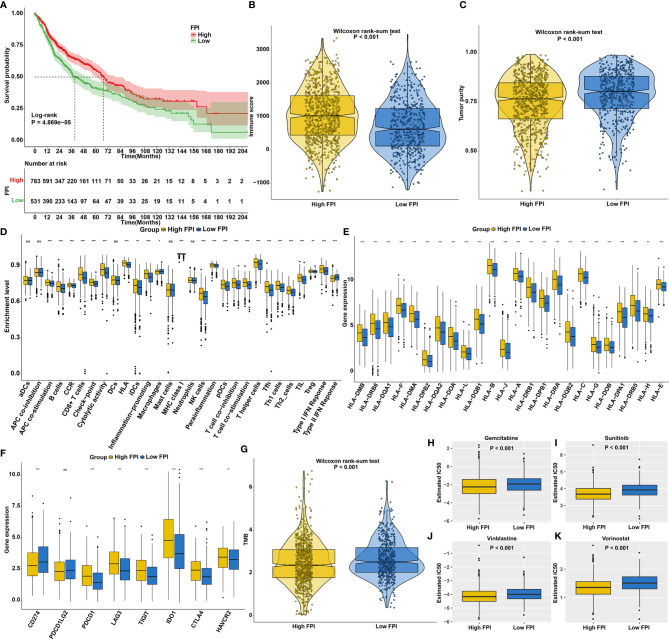
The roles of ferroptosis on prognosis, TME, response to immunotherapy and chemosensitivity in SCCs. **(A)** Kaplan-Meier curves of overall survival for SCCs patients with high and low FPI. P value was determined with log-rank test. **(B)** Differences in immune scores between high and low FPI groups. **(C)** Differences in tumor purity between high and low API groups. **(D)** Differences in the enrichment levels of immune cells and immune functions between two groups. **(E)** Differences in the expression levels of HLA genes between two groups. **(F)** Differences in the expression levels of immune checkpoints between two groups. **(G)** Differences in TMB scores between two groups. **(H–K)** Differences in estimated IC50 values of **(H)** Gemcitabine, **(I)** Sunitinib, **(J)** vinblastine and **(K)** vorinostat between two groups. Ns, not significant; *p<0.05; **p<0.01; ***p<0.001.

We first explored the prognostic significance of ferroptosis in SCCs. As anticipated, patients with high FPI exhibited prolonged survival duration (**Figure 5A**). High FPI was characterized by increased immune score (**Figure 5B**) and lowered tumor purity (**Figure 5C**). In **Figure 5D**, high FPI was significantly correlated to increased T-cell infiltration, increased IFN-γ response and decreased immunosuppressive cells (such as macrophages), which was present in immune-inflamed phenotype. Also, high FPI was characterized by increased HLA expression (**Figure 5E**) and increased immune checkpoint expression (**Figure 5F**). TMB is a predictive biomarker for identifying patients most likely to respond to immunotherapy (47). Low TMB was found in high FPI samples (**Figure 5G**). Further analysis revealed that high FPI patients were more sensitive to Gemcitabine (**Figure 5H**), Sunitinib (**Figure 5I**), Vinblastine (**Figure 5J**) and Vorinostat (**Figure 5K**).

### Synergistical Roles of Autophagy and Ferroptosis on Prognosis and Chemosensitivity of SCCs

**Figure 6:**
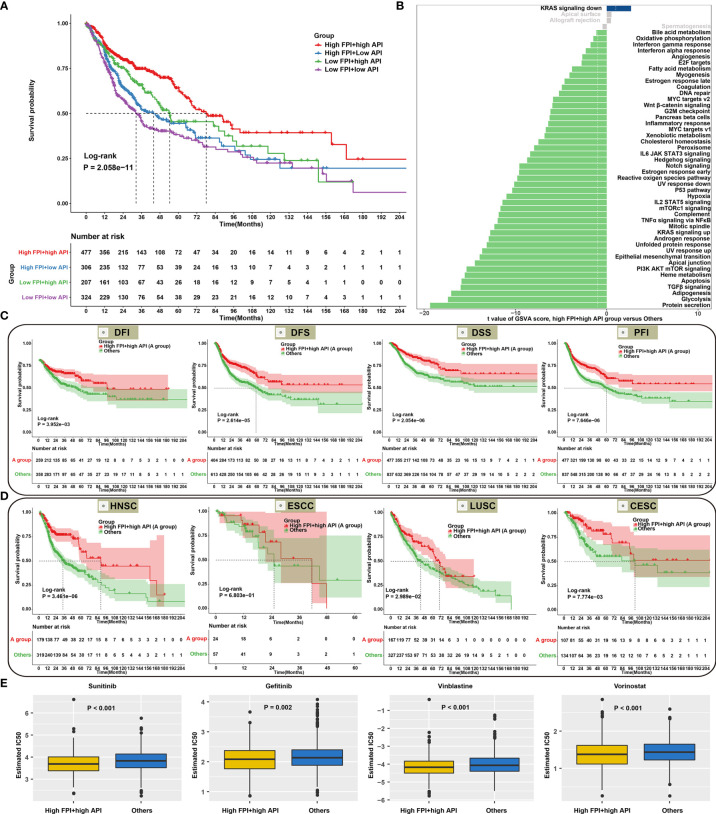
Synergistical roles of autophagy and ferroptosis on prognosis and chemosensitivity of SCCs. **(A)** Overall survival analysis for SCCs patients stratified by both API and FPI using Kaplan-Meier curves. P value was calculated with log-rank test. **(B)** Differences in signaling pathways between high FPI + high API group and “others” group in SCCs cohort. The “others” indicated the remaining patients with SCCs except for those with high FPI + high API. **(C)** Kaplan-Meier curves of DFI, DFS, DSS and PFI in patients with high FPI + high API and “others”. **(D)** Overall survival analysis for HNSC, ESCC, LUSC and CESC patients with high FPI + high API and “others”. **(E)** Differences in estimated IC50 values of Sunitinib, Gefitinib, Vinblastine and Vorinostat between two groups.

According to FPI and API scores, SCCs patients were stratified into four molecular patterns: high FPI + high API; high FPI + low API; low FPI + high API; low FPI + low API. Prognosis analysis revealed that patients with high FPI in concert with high API exhibited a prominent survival benefit (**Figure 6A**), indicating that autophagy and ferroptosis synergistically contributed to a favorable prognosis. GSVA was performed to better illustrate the biological behaviors of autophagy and ferroptosis. Surprisingly, carcinogenic pathways and immunity were remarkably enriched in high FPI and high API, indicating that the crosstalk of autophagy and ferroptosis played a nonnegligible role in ornamenting tumor immune microenvironment (**Figure 6B**; **Supplementary Table 6**). Further analysis showed that high FPI in concert with high API was linked to a better DFI, DFS, DSS and PFI of SCCs (**Figure 6C**). Moreover, we sought to determine the performance of the crosstalk of autophagy and ferroptosis in predicting OS outcomes in HNSC, ESCC, LUSC and CESC. As expected, patients with high FPI in concert with high API were remarkably correlated to a better prognosis in each SCC type (**Figure 6D**). We also observed that patients with high FPI in concert with high API were more sensitive to Sunitinib, Gefitinib, Vinblastine and Vorinostat (**Figure 6E**). Therefore, the crosstalk of autophagy and ferroptosis was significantly relevant to SCCs progression, recurrence, and chemotherapy resistance.

### Synergistical Roles of Autophagy and Ferroptosis Shape an Inflamed TME

**Figure 7:**
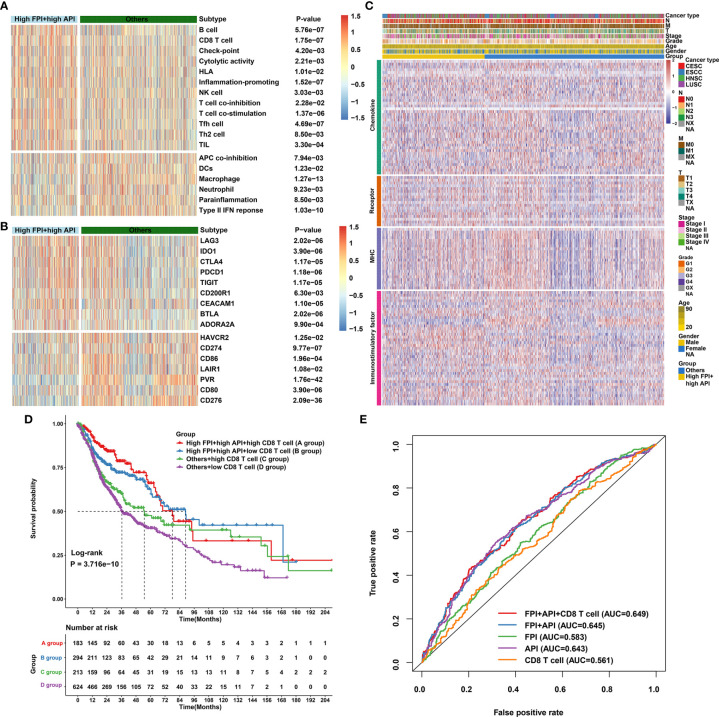
Synergistical roles of autophagy and ferroptosis on immunity and survival outcomes of SCCs. **(A)** Heatmap showing the differences in immune cell infiltrations and immune functions in patients with high FPI + high API group and “others” group. The “others” indicated the remaining patients with SCCs except for those with high FPI + high API. **(B)** Heatmap of the expression levels of immune checkpoints in patients with high FPI + high API group and “others”. **(C)** Differences in the expression levels of 122 immunomodulators (chemokines, receptors, MHC, and immunostimulatory factors) between high FPI + high API and “others” groups in SCCs. **(D)** Overall survival analysis for SCCs patients stratified by API, FPI and CD8^+^ T cells using Kaplan-Meier curves. P value was determined with log-rank test. **(E)** Predictive accuracy of API, FPI, CD8^+^ T cells or combinations according to the area under the ROC curves.

High FPI and high API SCCs samples showed increased T-cell infiltrations (such as CD8^+^ T cell, Tfh cell, Th2 cell and TIL) and low immunosuppressive cell populations (such as macrophages; **Figure 7A**), indicating that crosstalk between autophagy and ferroptosis might be involved in modulating immune cell infiltration. Most immune checkpoints (LAG3, IDO1, CTLA4, PD-1, TIGIT, CD200R1, CEACAM1, BTLA and ADORA2A) were found to be up-regulated in high FPI and high API samples (**Figure 7B**). Furthermore, our findings revealed that high FPI in concert with high API was positively associated with a majority of immunomodulators in SCCs (**Figure 7C**). Thus, synergistical roles of autophagy and ferroptosis might shape an inflamed TME of SCCs. Antitumor immunity is mediated to a large extent by CD8^+^ T cells. Emerging evidence suggests that autophagy and ferroptosis changes in CD8^+^ T cell metabolism directly modulate anti-tumor immunity (48, 49). Hence, it is of significance to comprehensively analyze the synergistical roles of autophagy, ferroptosis and CD8^+^ T cell infiltration on SCC prognosis. Combining CD8^+^ T cells, we found that patients with low infiltration of CD8^+^ T cells and “others” experienced the worst clinical outcomes (**Figure 7D**). The predictive efficacy of CD8^+^ T cell, API, FPI and their combinations was evaluated by ROC analysis. In **Figure 7E**, combination of CD8^+^ T cells, API, FPI exhibited the best performance on predicting SCCs prognosis (**Supplementary Figure 6**).

**Figure 8:**
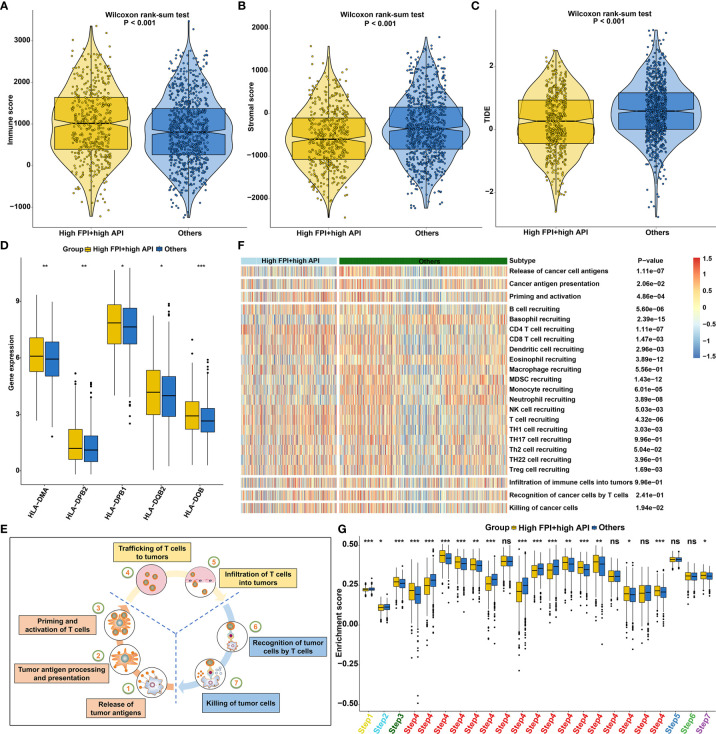
Synergistical roles of autophagy and ferroptosis shape an inflamed TME of SCCs. **(A–C)** Differences in **(A)** immune score, **(B)** stromal score and **(C)** tumor purity between high FPI + high API and “others” groups in SCCs. The “others” indicated the remaining patients with SCCs except for those with high FPI + high API. **(D)** Differences in the expression of HLA genes between patients with high FPI + high API and “others”. **(E)** Schematic diagram of the cancer immunity cycle. **(F, G)** Differences in the activity of the steps of the cancer immunity cycle between high FPI + high API and “others” groups in SCCs. Ns, not significant; *p<0.05; **p<0.01; ***p<0.001.

ESTIMATE algorithm was employed to quantify the overall infiltration of immune cells and stromal cells in SCCs tissue. Our results showed high FPI and high API samples were characterized by increased immune score (**Figure 8A**) and low stromal score (**Figure 8B**), which also confirmed that the crosstalk of autophagy and ferroptosis was linked to immune cell infiltrations. Furthermore, we found that tumors with high FPI in concert with high API exhibited low TIDE scores (**Figure 8C**). This indicated that the crosstalk of autophagy and ferroptosis might influence response to immune checkpoint blockade for SCCs patients. High expression of HLAs (HLA-DMA, HLA-DPB2, HLA-DPB1, HLA-DQB2 and HLA-DOB) was found in SCCs with high FPI in concert with high API (**Figure 8D**). The activities of the cancer immunity cycle are the direct comprehensive performance of the functions of the chemokine system and other immunomodulators (**Figure 8E**). For specimens with high FPI in concert with high API, activities of most of the steps in the cycle were found to be up-regulated, including priming and activation (step 3), B cell recruiting (step 4), CD4+ T cell recruiting (step 4), CD8^+^ T cell recruiting (step 4), dendritic cell recruiting (step 4), NK cell recruiting (step 4), T cell recruiting (step 4), Th1 cell recruiting (step 4), Th2 cell recruiting (step 4), Treg cell recruiting (step 4) and killing of cancer cells (step 7; **Figures 8F, G**). These data indicated that synergistical roles of autophagy and ferroptosis might shape an inflamed TME in SCCs.

### Bioactive Compounds for SCCs Treatment Based on Autophagy- and Ferroptosis-Related Genes

**Figure 9:**
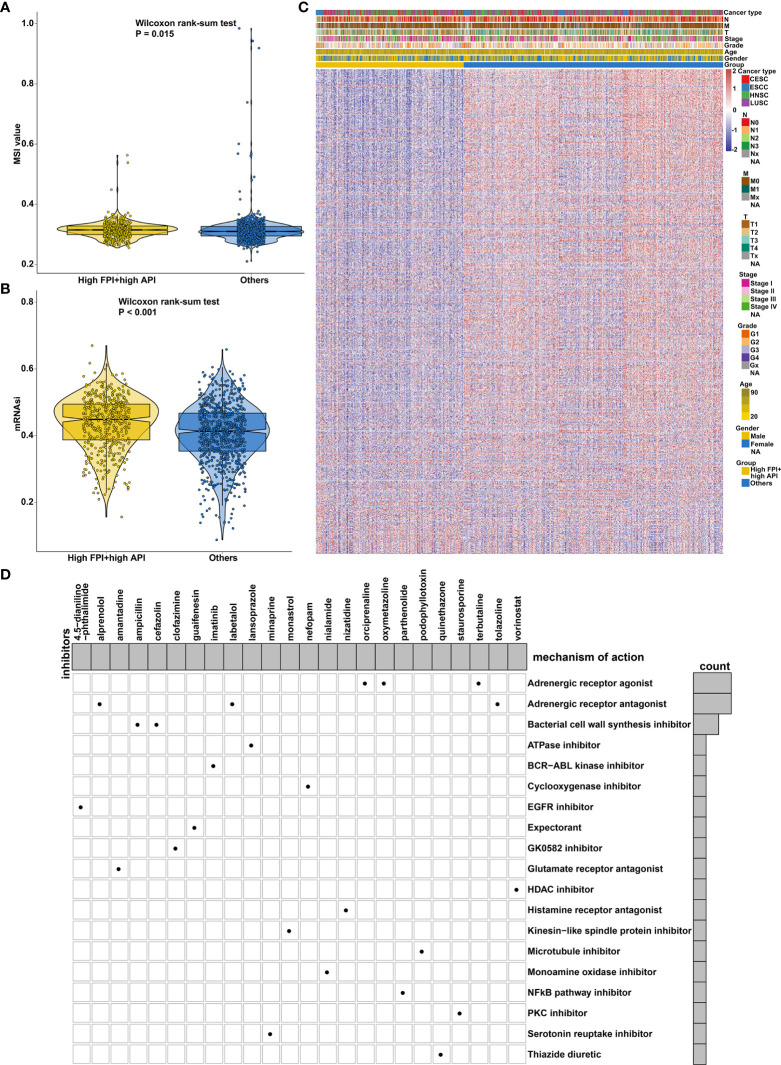
Synergistical roles of autophagy and ferroptosis on MSI status and cancer stemness and prediction of potential bioactive compounds for SCCs treatment. **(A)** Differences in MSI status between high FPI + high API and “others” groups in SCCs. **(B)** Differences in mRNAsi between groups in SCCs. **(C)** Heatmap of the expression of DEGs between high FPI + high API and “others” groups. Red indicated high expression and blue indicated low expression. The gender, age, grade, stage, T, N, M and SCCs type were used as patient annotations. **(D)** Heatmap for each compound (perturbagen) from the CMap that shared a MoA (rows) based on autophagy- and ferroptosis-related genes, ranked by descending number of compounds with a shared MoA.

Mismatch repair deficiency (dMMR) leads to microsatellite instability (MSI), which is in relation to response to immune- and chemotherapies (50). We found that there was a distinct difference in MSI status between high FPI + high API group and “others” group (**Figure 9A**). Cancer stem cells (CSCs) are characterized by differentiation, self-renewal, and homeostatic control, which allowing tumor maintenance and spread. Increasing evidence has demonstrated that recurrence and therapeutic resistance of SCCs are attributed to CSCs (51). Here, this study quantified cancer stemness by mRNAsi in SCCs. Increased mRNAsi was found in SCCs specimens with high FPI in concert with high API (**Figure 9B**). To further observe the potential biological behaviors of the crosstalk between autophagy and ferroptosis, we identified 154 down- and 538 up-regulated genes in high FPI + high API group compared to “others” group (**Figure 9C**; **Supplementary Table 7**). Potential drugs for SCCs treatment were predicted by CMap based on these up and down-regulated tags, respectively. Following the signature query, arecoline, ketotifen and viomycin with the highest positive enrichment score were determined as potential bioactive compounds for specifically activating autophagy and ferroptosis (**Supplementary Table 8**). MoA analysis of predicted compounds demonstrated mechanisms of action shared by the compounds (**Figure 9D**). Three compounds (orciprenaline, oxymetazoline and terbutaline) shared the MoA of adrenergic receptor agonist and three compounds (oxprenolol, labetalol and tolazoline) shared adrenergic receptor antagonist.

### Generation of a Prognostic Model Combining Autophagy and Ferroptosis

**Figure 10:**
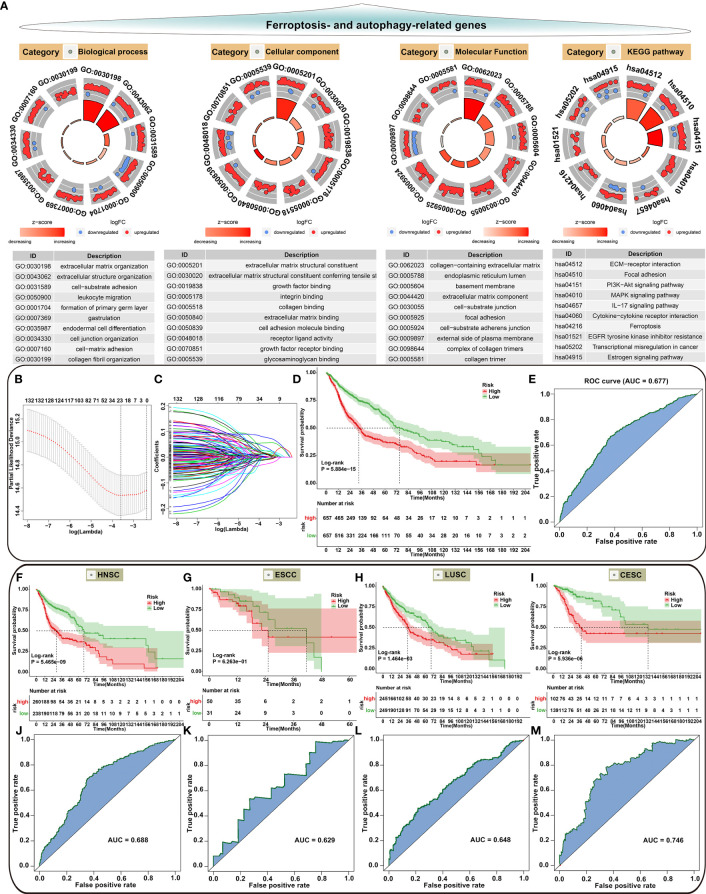
Biological functions of autophagy- and ferroptosis-related genes and development of a prognostic model for SCCs. **(A)** Functional annotation analysis showing the biological functions and pathways involving autophagy- and ferroptosis-related genes. **(B)** Cross-validation for turning parameter selection by the minimum criteria in the LASSO regression model. Two dotted vertical lines were depicted at the optimal values based on the minimum criteria. Totally, 22 optimal DEGs with the best discriminative ability were selected for establishing the model. **(C)** LASSO coefficient profiles of 138 prognostic DEGs in SCCs. The coefficient profiles were plotted according to the log (Lambda) values. **(D)** Kaplan-Meier curves of overall survival for patients with high and low risk. P value was determined with log-rank test. **(E)** Assessment of the predictive accuracy of the model for survival of SCCs patients from TCGA cohort according to the area under ROC curves. **(F–I)** Survival analysis for the two groups in **(F)** HNSC, **(G)** ESCC, **(H)** LUSC and **(I)** CESC patients from TCGA cohort. **(J–M)** Predictive accuracy of the model for survival of **(J)** HNSC, **(K)** ESCC, **(L)** LUSC and **(M)** CESC patients.

**Figure 11:**
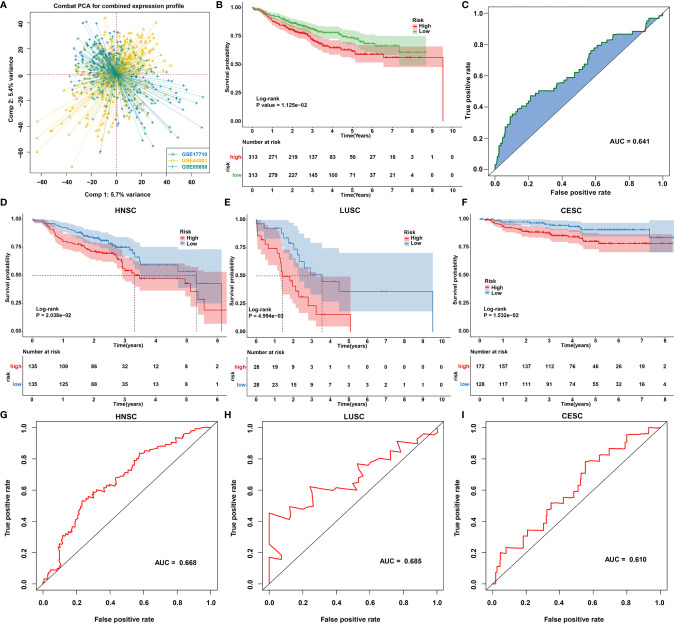
Validation of the autophagy- and ferroptosis-related prognostic model for SCCs in external cohorts. **(A)** Principal component analysis showing the batch effects removed for three external cohorts: GSE17710, GSE44001 and GSE65858. **(B, C)** Validation of the predictive accuracy of the model for SCCs prognosis using integrated three datasets. **(D–F)** Validation of the overall survival of **(D)** HNSC, **(E)** LUSC and **(F)** CESC patients with high and low risk using Kaplan-Meier curves. **(G–I)** Validation of the predictive efficacy of the model for **(G)** HNSC, **(H)** LUSC and **(I)** CESC patients based on the area under ROC curves.

Our functional enrichment analysis showed that autophagy- and ferroptosis-related genes were significantly enriched in extracellular matrix (ECM), cancer-related pathways (PI3K-Akt signaling pathway, MAPK signaling pathway, EGFR tyrosine kinase inhibitor resistance, transcriptional misregulation in cancer and estrogen signaling pathway) and ferroptosis (**Figure 10A**), indicating the potential clinical implications of these genes. Among all autophagy- and ferroptosis-related genes, 138 were significantly associated with prognosis of SCCs patients using univariate Cox regression analysis (**Supplementary Table 9**). With the LASSO Cox regression method, 22 optimal candidate genes were selected with the minimum lambda (**Figures 10B, C**). A risk score model was created based on the expression and coefficients of the candidate genes (**Supplementary Table 10**). In TCGA cohort, SCCs patients were stratified into high-risk group (n=657) and low-risk group (n=657) with the median risk score as the cutoff value. In **Figure 10D**, patients with high risk indicated worse OS time compared to those with low risk. The AUC of the risk score was 0.677 (**Figure 10E**). We further investigated the prognostic value of the risk score in each SCC type. High risk scores were distinctly correlated to poorer prognosis for HNSC (**Figure 10F**), ESCC (**Figure 10G**), LUSC (**Figure 10H**) and CESC (**Figure 10I**). The well predictive performance was also observed in each SCC type (**Figures 10J**–**M**). After removing batch effects (**Figure 11A**), three external datasets (GSE17710, GSE44001 and GSE65858) were employed to confirm the excellent predictive accuracy of the risk score in SCCs prognosis (**Figures 11B, C**). Furthermore, we found that the risk score was a promising prognostic panel for each SCC type (**Figures 11D**–**I**).

### Synergistical Roles of Autophagy and Ferroptosis on Gefitinib Sensitivity and Tumor Progression in SCCs

**Figure 12:**
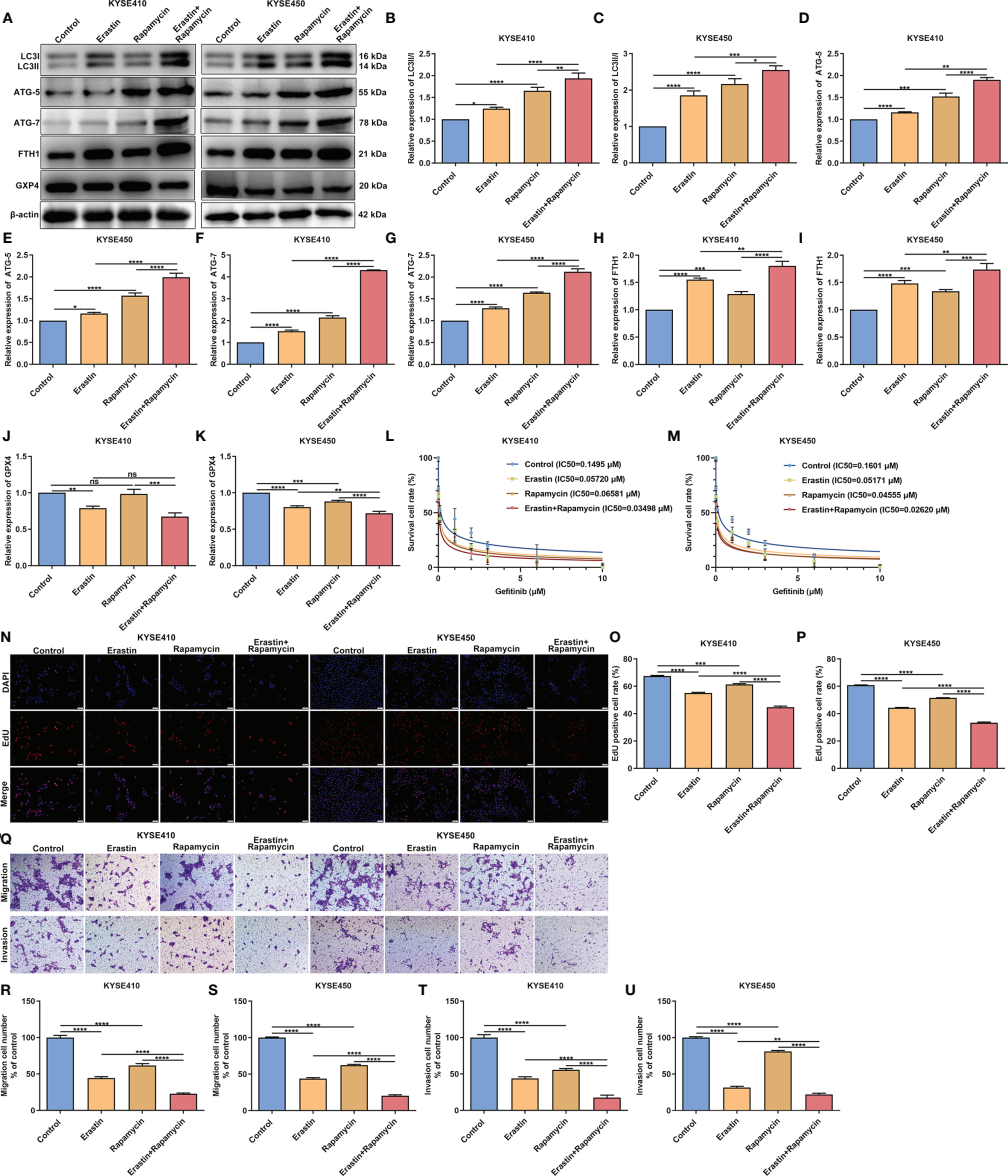
Synergistical roles of autophagy and ferroptosis on gefitinib sensitivity and tumor progression in SCCs. **(A–K)** Detection of the expression of autophagy-related proteins including LC3II/I, ATG-5 and ATG-7 as well as ferroptosis-related proteins including FTH1 and GPX4 in KYSE410 and KYSE450 SCC cells exposed to Erastin and/or Rapamycin through western blotting. **(L, M)** Cell viability of KYSE410 and KYSE450 cells under treatment with Erastin and/or Rapamycin by MTT assay. **(N–P)** Measurement of proliferation of KYSE410 and KYSE450 cells treated with Erastin and/or Rapamycin by EdU staining. Scale bar, 50 μm and magnification, 200×. **(Q–U)** Detection of migration and invasion of KYSE410 and KYSE450 cells following administration with Erastin and/or Rapamycin using transwell assay. Scale bar, 50 μm and magnification, 200×. Ns, not significant; *p<0.05; **p<0.01; ***p<0.001; ****p<0.0001.

We further validated the synergistical roles of autophagy and ferroptosis on gefitinib resistance and tumor progression in SCCs through *in vitro* experiments. Two SCC cell lines KYSE410 and KYSE450 were exposed to ferroptosis agonist Erastin and autophagy agonist Rapamycin to confirm the crosstalk between autophagy and ferroptosis in SCCs. Our western blotting results showed that both Erastin and Rapamycin significantly enhanced the expression of autophagy-related proteins including LC3II/I (**Figures 12A**–**C**), ATG-3 (**Figures 12D, E**) and ATG-3 (**Figures 12F, G**). Additionally, both Erastin and Rapamycin significantly increased the expression of ferroptosis-related protein FTG1 (**Figures 12H, I**) but reduced the expression of ferroptosis inhibitor GPX4 (**Figures 12J, K**). As expected, co-treatment of Erastin and Rapamycin synergistically prominently enhanced autophagy and ferroptosis in KYSE410 and KYSE450 cells. Our cell viability assays demonstrated that both Erastin and Rapamycin reduced the gefitinib IC50 values than control cells (**Figures 12L, M**). Additionally, we observed the synergistical roles of Erastin in concert with Rapamycin on gefitinib sensitivity. EdU staining (**Figures 12N**–**P**) and transwell (**Figures 12Q**–**U**) assays demonstrated that proliferation, migration and invasion were remarkedly suppressed by Erastin or Rapamycin in KYSE410 and KYSE450 cells. Also, there were synergistical roles of Erastin and Rapamycin on inhibiting proliferation, migration and invasion of KYSE410 and KYSE450 cells. Above evidence confirmed the synergistical roles of autophagy and ferroptosis on gefitinib sensitivity and tumor progression in SCCs.

## Discussion

Autophagy, a lysosome-dependent catabolic process, promotes cell survival and accelerates cellular demise (12). Ferroptosis, an iron-dependent cell death type, is in relation to the accumulation of lethal reactive lipid species (52). Recent experiment findings indicate that ferroptosis could occur while sharing common pathways or regulators with autophagy (15). Consistent with published research, this study comprehensively uncovered the close crosstalk between autophagy and ferroptosis at the molecular level (18). Moreover, their interplay was closely related to TME, immunity, chemotherapy resistance and survival outcomes of SCCs.

Herein, we quantified FPI and API for reflecting ferroptosis and autophagy levels in SCCs *via* PCA method. Their synergistical roles contributed to favorable survival outcomes of SCCs. The TME that is mainly composed of cancer cells, immune cells, and other components may mediate SCCs development and therapeutic response (53). Novel anticancer therapeutic strategies are required to target the pathways and molecular communications between cancer cells and the surrounding immune cells in the TME (54). Although previous experiment findings have reported the interplay between autophagy, ferroptosis and anti-tumor immunity, there is still lack of evidence from human SCCs specimens that may hinder the clinical translation (16). We found that autophagy in concert with ferroptosis participated in shaping an inflamed TME in human SCCs. Immune cells such as CD8^+^ T cells are related to favorable prognosis of patients and increased curative effects of immunotherapy (55). Herein, we found that high CD8^+^ T cell infiltration in concert with high FPI and high API indicated undesirable survival outcomes of SCCs and their combination displayed the well predictive efficacy in SCCs prognosis. Recent experiments have reported that CD8^+^ T cells may inhibit tumor growth through inducing ferroptosis and autophagy (48). ICI therapy may be combined with other strategies that transform “cold tumors” to “hot tumors”, which may increase sensitivity to ICI therapy. Tumors usually induce immune checkpoint expression for avoid being detected and killed by the host immune system (56). Therapies with anti-PD-1, anti-PD-L1, or anti-CTLA-4 reinvigorate T cells as well as allow the adaptive immune system thereby targeting cancer cells. Our data indicated that the induction of ferroptosis and autophagy combined with ICIs might produce synergistically enhanced antitumor activity for SCCs. Resistance to chemotherapy and molecular targeted therapies is a major problem facing current cancer research, which severely limits the effectiveness of cancer therapies. We found that synergistical roles of autophagy and ferroptosis may improve the sensitivity to sunitinib, gefitinib, vinblastine and vorinostat for SCCs patients. Taken together, the induction of autophagy and ferroptosis combined with immune- or chemotherapies might produce synergistically enhanced anti-SCCs activity. By CMap database, this study predicted arecoline, ketotifen and viomycin with the highest positive enrichment score as potential small molecule compounds for specifically activating autophagy and ferroptosis. Our *in vitro* experiments showed that ferroptosis agonist Erastin and autophagy agonist Rapamycin synergistically enhanced the sensitivity to gefitinib and suppressed cell proliferation, migration and invasion in SCCs cells, indicating the synergistical roles of autophagy and ferroptosis on gefitinib sensitivity and tumor progression in SCCs.

To facilitate personalized prediction of the prognosis of SCCs patients, we established a prognostic model based on 22 autophagy- and ferroptosis-related genes utilizing the LASSO algorithm to improve predictive accuracy for SCCs. Following external verification, this prognostic model possessed the well performance in predicting patients’ prognosis. Nevertheless, this model will be validated in a prospective cohort.

## Conclusion

Collectively, our bioinformatic analysis uncovered the interplay between autophagy and ferroptosis and their synergistical roles on prognosis, TME, immunity, and chemotherapy resistance in SCCs. The concomitant induction of autophagy and ferroptosis may be a promising strategy for treating SCCs. Although we predicted several bioactive compounds, potent drugs that function in activating autophagy and ferroptosis should be designed in future studies. Furthermore, clinical trials that treat patients with approved drugs that specifically activate autophagy and ferroptosis with the concomitant utilization of ICIs will be carried out in the future.

## Data Availability Statement

The datasets presented in this study can be found in online repositories. The names of the repository/repositories and accession number(s) can be found in the article/**Supplementary Material**.

## Author Contributions

MZ conceived and designed the study. LC, XN, and XQ conducted most of the experiments and data analysis, and wrote the manuscript. SL, HM, XS, and XH participated in collecting data and helped to draft the manuscript. All authors contributed to the article and approved the submitted version.

## Funding

This work was funded by the National Natural Science Foundation of China (81072197, 81470758).

## Conflict of Interest

The authors declare that the research was conducted in the absence of any commercial or financial relationships that could be construed as a potential conflict of interest.

## Publisher’s Note

All claims expressed in this article are solely those of the authors and do not necessarily represent those of their affiliated organizations, or those of the publisher, the editors and the reviewers. Any product that may be evaluated in this article, or claim that may be made by its manufacturer, is not guaranteed or endorsed by the publisher.

## Supplementary Material

Supplementary Table 1 Summary of autophagy- and ferroptosis-related genes.

Supplementary Table 2 The list of HLA genes, immune checkpoints and immunomodulators.

Supplementary Table 3 The cancer immunity cycle.

Supplementary Table 4 The API scores of SCCs samples.

Supplementary Table 5 The FPI scores of SCCs samples.

Supplementary Table 6 The activated signaling pathways in high FPI and high API SCCs samples by GSVA.

Supplementary Table 7 Differentially expressed genes associated with high FPI and high API SCCs.

Supplementary Table 8 Bioactive compounds for SCCs treatment based on autophagy- and ferroptosis-related genes.

Supplementary Table 9 Autophagy- and ferroptosis-related genes that could impact survival of SCCs patients using univariate cox regression analysis.

Supplementary Table 10 Coefficients of 22 autophagy- and ferroptosis-related genes in the risk score model for SCCs.

Supplementary Figure 1 Correction of batch effects of HNSC, LUSC, CESC and ESCC datasets from TCGA database.

Supplementary Figure 2 The somatic mutations of **(A)** autophagy- and **(B)** ferroptosis-related genes in HNSC, ESCC, LUSC and CESC samples.

Supplementary Figure 3 The autophagy-related genes that could significantly impact the survival of **(A)** HNSC, **(B)** ESCC, **(C)** LUSC and **(D)** CESC patients.

Supplementary Figure 4 The ferroptosis-related genes that could significantly impact the survival of **(A)** HNSC, **(B)** ESCC, **(C)** LUSC and **(D)** CESC patients.

Supplementary Figure 5
**(A)** The autophagy- and **(B)** ferroptosis-related genes that could significantly impact the survival of SCCs patients.

Supplementary Figure 6 Predictive accuracy of API, FPI, CD8^+^ T cells or combination for **(A)** HNSC, **(B)** ESCC, **(C)** LUSC and **(D)** CESC prognosis according to the area under the ROC curves.

## Abbreviations

TME, tumor microenvironment; SCCs, squamous cell carcinomas; TCGA, the Cancer Genome Atlas; ICIs, immune checkpoint inhibitors; HNSC, head and neck squamous cell carcinoma; LUSC, lung squamous cell carcinoma; CESC, cervical squamous cell carcinoma; ESCC, esophageal squamous cell carcinoma; CNV, copy number variation; PCA, principal component analysis; FPI, ferroptosis potential index; API, autophagy potential index; PPI, protein-protein interaction; OS, overall survival; DFI, disease-free interval; DFS, disease-free survival; DSS, disease-specific survival; PFI, progression-free interval; ROC, receiver-operating characteristic; AUC, area under the ROC curve; ESTIMATE, Estimation of STromal and Immune cells in MAlignant Tumours using Expression data; ssGSEA, single-sample gene-set enrichment analysis; HLA, human leukocyte antigen; TIDE, T cell dysfunction and exclusion; TMB, tumor mutation burden; GDSC, Genomics of Drug Sensitivity in Cancer; IC50, half-maximal inhibitory concentration; GSVA, gene set variation analysis; mRNAsi, mRNA expression-based stemness index; FDR, false discovery rate; GO, Gene Ontology; KEGG, Kyoto Encyclopedia of Genes and Genomes; CMap, Connectivity Map; MoA, mode of action; LASSO, least absolute shrinkage and selection operator; dMMR, mismatch repair deficiency; CSCs, Cancer stem cells; MTT, 3-[4,5-dimethylthiazol-2-yl]-2, 5-diphenyltetrazoliumbromide; EdU, Ethynyl-2’-deoxyuridine.
