# Supplementary figures and images for: Characterization of Interplay Between Autophagy and Ferroptosis and Their Synergistical Roles on Manipulating Immunological Tumor Microenvironment in Squamous Cell Carcinomas
Source: Front Immunol. 2022 Feb 4;12:739039. doi: 10.3389/fimmu.2021.739039 (PMC8854375; doi:10.3389/fimmu.2021.739039)

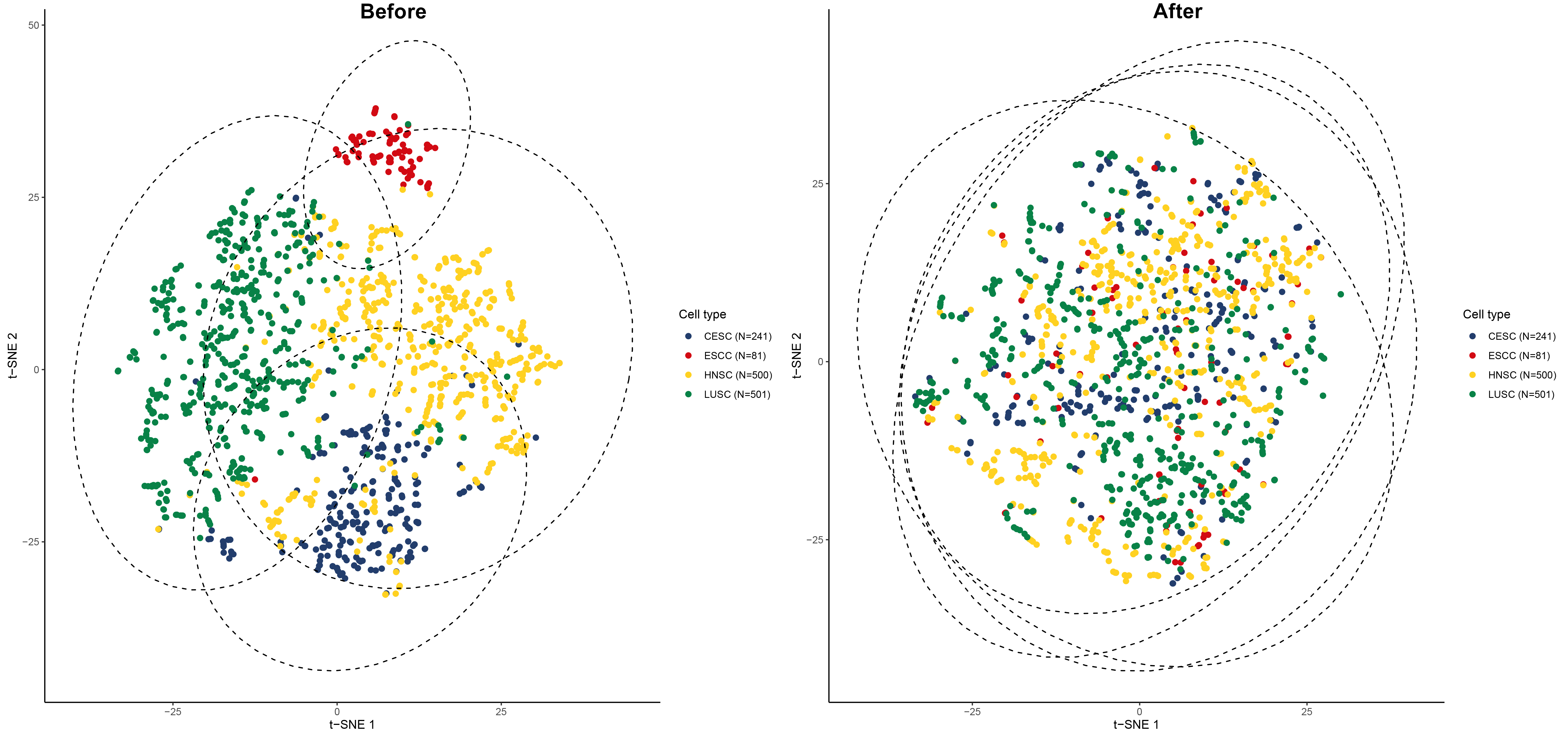

Supplement: Supplementary Figure 1 — Correction of batch effects of HNSC, LUSC, CESC and ESCC datasets from TCGA database. [file Image_1.tif]

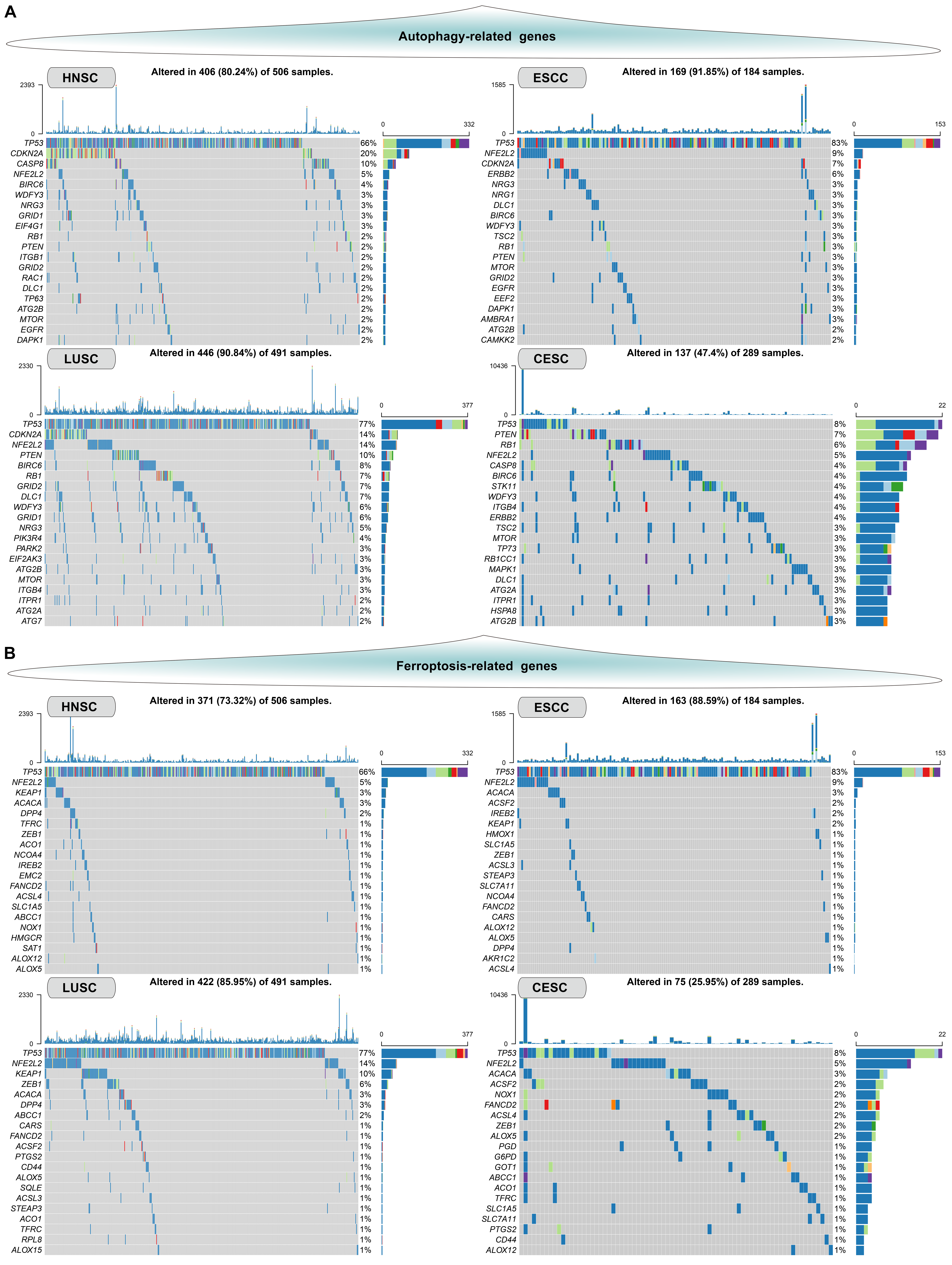

Supplement: Supplementary Figure 2 — The somatic mutations of (A) autophagy- and (B) ferroptosis-related genes in HNSC, ESCC, LUSC and CESC samples. [file Image_2.tif]

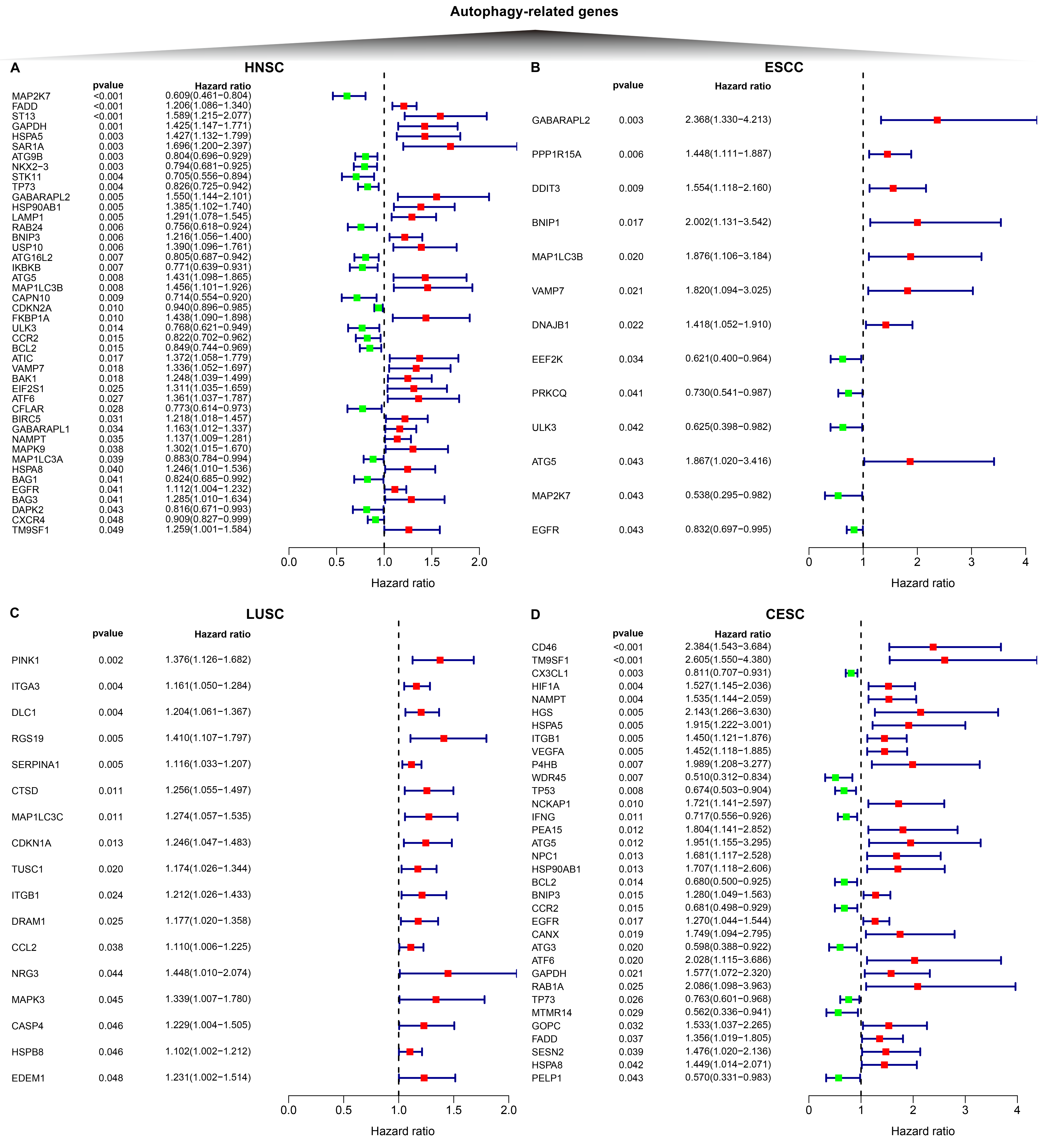

Supplement: Supplementary Figure 3 — The autophagy-related genes that could significantly impact the survival of (A) HNSC, (B) ESCC, (C) LUSC and (D) CESC patients. [file Image_3.tif]

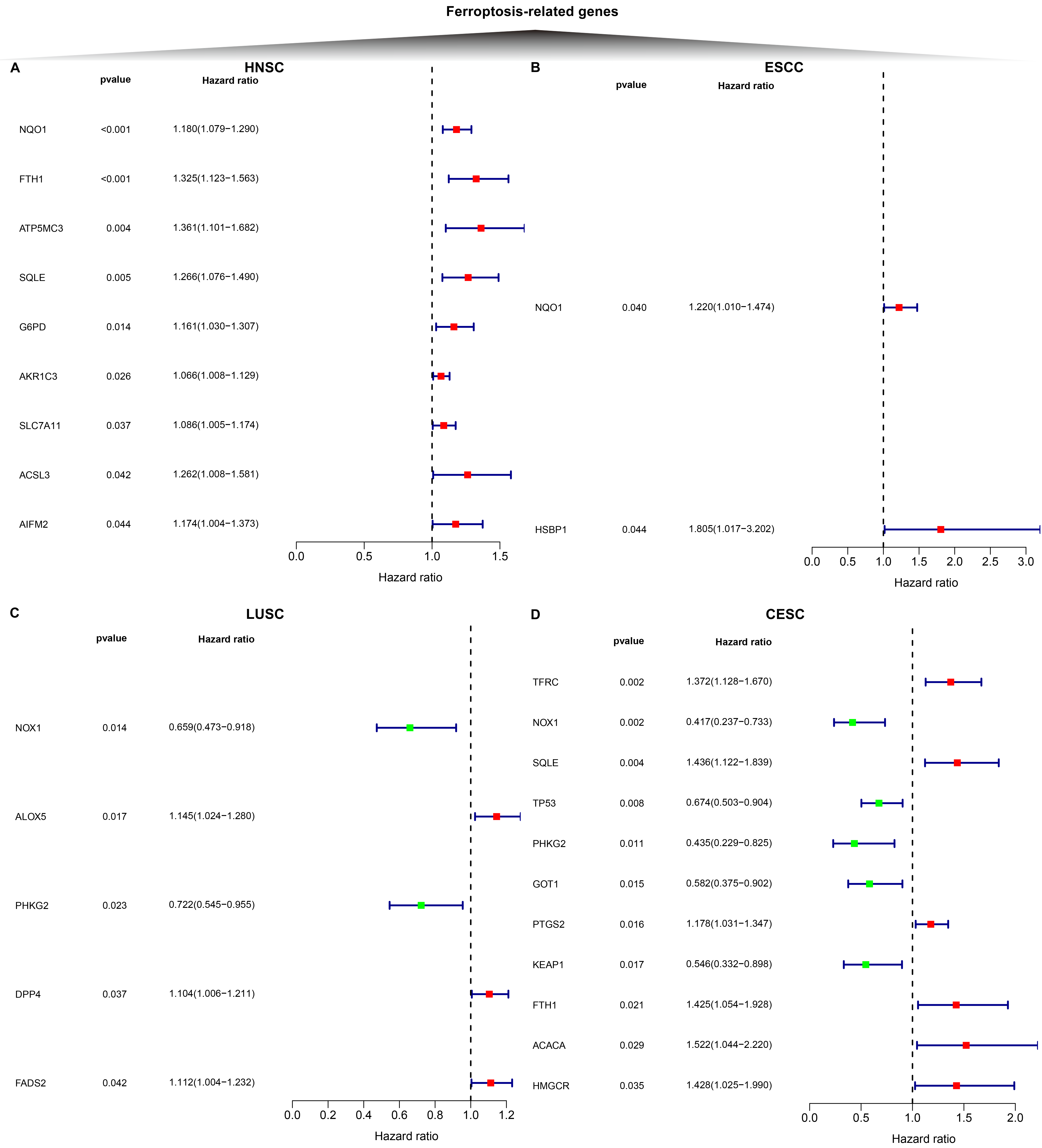

Supplement: Supplementary Figure 4 — The ferroptosis-related genes that could significantly impact the survival of (A) HNSC, (B) ESCC, (C) LUSC and (D) CESC patients. [file Image_4.tif]

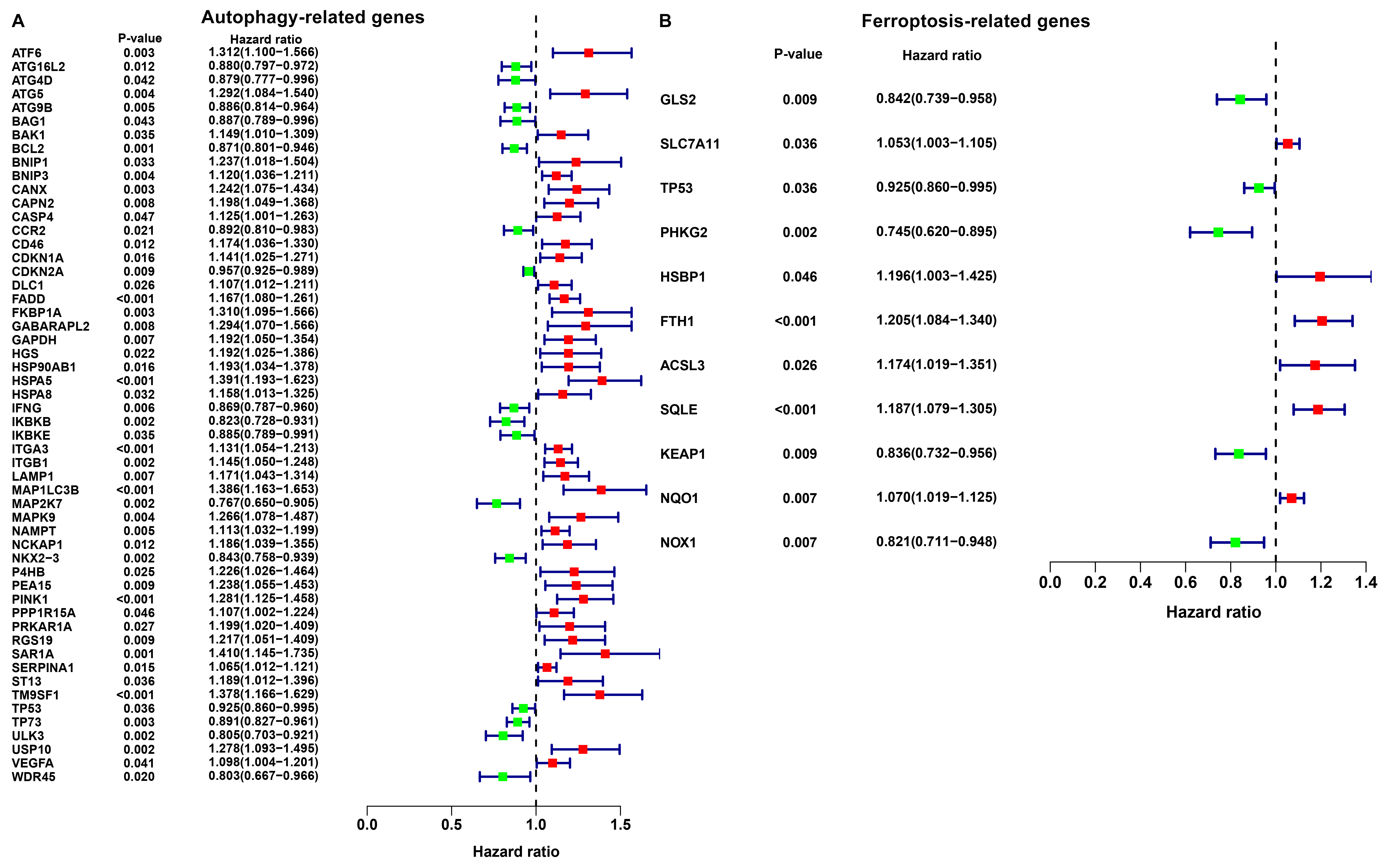

Supplement: Supplementary Figure 5 — (A) The autophagy- and (B) ferroptosis-related genes that could significantly impact the survival of SCCs patients. [file Image_5.tif]

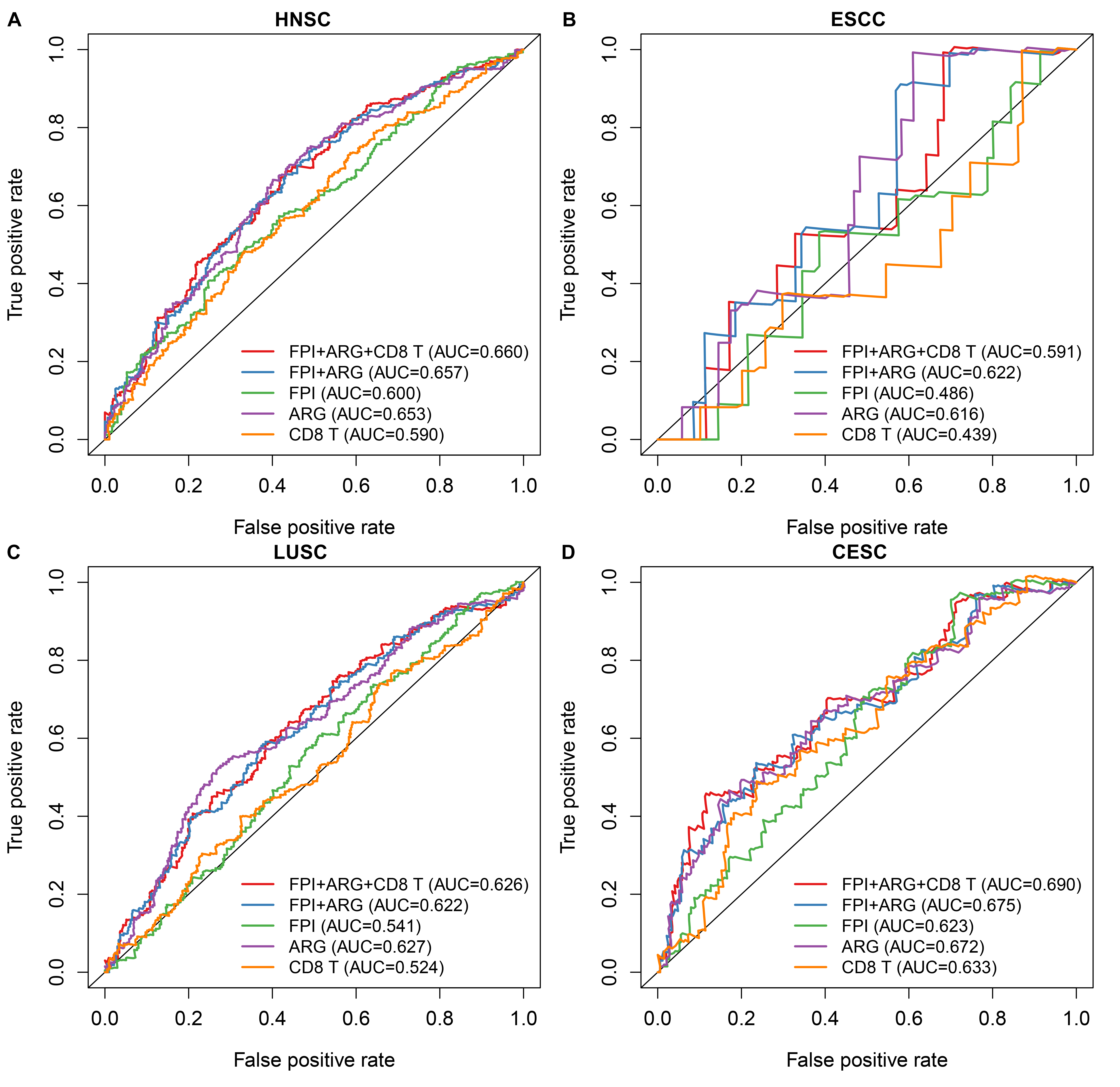

Supplement: Supplementary Figure 6 — Predictive accuracy of API, FPI, CD8+ T cells or combination for (A) HNSC, (B) ESCC, (C) LUSC and (D) CESC prognosis according to the area under the ROC curves. [file Image_6.tif]
